# TestSTORM: Versatile simulator software for multimodal super-resolution localization fluorescence microscopy

**DOI:** 10.1038/s41598-017-01122-7

**Published:** 2017-04-19

**Authors:** Tibor Novák, Tamás Gajdos, József Sinkó, Gábor Szabó, Miklós Erdélyi

**Affiliations:** 1 Department of Optics and Quantum Electronics, University of Szeged, Dóm tér 9, 6720 Szeged, Hungary; 2 MTA-SZTE Research Group on Photoacoustic Spectroscopy, Szeged, Hungary

## Abstract

Optimization of sample, imaging and data processing parameters is an essential task in localization based super-resolution microscopy, where the final image quality strongly depends on the imaging of single isolated fluorescent molecules. A computational solution that uses a simulator software for the generation of test data stacks was proposed, developed and tested. The implemented advanced physical models such as scalar and vector based point spread functions, polarization sensitive detection, drift, spectral crosstalk, structured background etc., made the simulation results more realistic and helped us interpret the final super-resolved images and distinguish between real structures and imaging artefacts.

## Introduction

Optical super-resolution techniques such as single molecule localization (SMLM), stimulated emission depletion (STED) and structured illumination (SIM) have become one of the most dynamically developed areas in optical microscopy^1–3^. These techniques routinely provide images of fixed cells or tissues with sub-diffraction spatial resolution (<250 nm), and can be applied even for live cell imaging under appropriate experimental conditions^4^. Localization techniques such as stochastic optical reconstruction microscopy (STORM), direct stochastic optical reconstruction microscopy ((d)STORM) and photo-activated localization microscopy (PALM) are based on the precise fitting of the point spread function (PSF) to the measured images of stochastically excited individual fluorescent molecules. These techniques require a tight control of rates between the on, off and the bleached states, keeping the number of active fluorescent molecules at an optimum value, so their diffraction limited images can be detected separately both spatially and temporally. This condition can be achieved by using switching buffers (dSTORM, STORM)^5, 6^, photoactivatable fluorescent proteins (PALM)^7, 8^ or dyes that can activate upon binding to the target molecules or structures^9^. The number of detected photons emitted by a single molecule determines the localization precision: the more photons are captured, the higher localization precision can be achieved^10^. However, the number of both the emitted photons and the switching circles of a single fluorescent molecule strongly depend on the dye and its local environment^6^. The most widely used dyes for dSTORM imaging, such as ATTO 647 and Alexa Fluor 647, can emit 4–5 thousands photons during a single switching event and can be activated 10–30 times sequentially before they are finally photobleached^6^. It means that such molecules can be localized 0–30 times, resulting in a scattered spot of imprecise localizations instead of a single highly localized dot. The size of these extended spots depends on the length of the linker, the flexibility of the bonds, the speed of rotation around the epitope, the local viscosity, the localization precision and even on the relative orientation of the dye molecule to the polarization of the excitation light. Because of the numerous (and sometimes unknown) parameters, the system can be handled only stochastically. For example, the quick rotation of the dye molecules obscures the polarization dependent PSF shape, and only an averaged distribution, typically estimated by a Gaussian function, can be observed. However, additional optical properties (polarization, spectra etc.)^11, 12^ of the emitted photons can be used to further monitor the chemical and physical properties (viscosity, pH, etc.) of the local environment. Combination of these features with multicolour imaging is a challenge and requires parameter optimization of the sample, the image acquisition and data processing, respectively. Simulations using different test samples such as the Siemens star pattern^13^, helix and deformed spiders^14^, alternating stripes with variable width^15^, isolated parallel and crossing lines and arrays^16, 17^ have been published. These test samples were specifically designed and applied for demonstration purposes. To our knowledge TestSTORM 1.0 software was the first code directly developed to generate image stacks for conventional localization microscopes, where localization meant the precise determination of the spatial (2D or 3D) position of the molecules^18^. In the meantime localization techniques have developed considerably and moved into the multimodality (spectral, polarization sensitive detections) direction^11, 12, 19^. This paper presents a significantly improved TestSTORM code with several new features such as scalar and vector diffraction based PSF models, drift, structured background, multicolour imaging and polarization sensitive excitation and detection. The generated image stacks are evaluated by rainSTORM^20^ throughout the paper. RainSTORM was also developed by the contribution of our group and has some unique features tested in this paper. TestSTORM and rainSTORM codes work sequentially but independently, clearly separating the data acquisition and the localization steps.

An open source simulation software called super-resolution simulator (SuReSim^21^) has been developed recently. SuReSim can be used for simulation of 2D and 3D structures (epitopes, lines and surfaces) based on ground truth models. The generated TIFF stacks can represent raw date acquired by a STORM microscope system. Several sample parameters (labelling efficiency, binding angle, number of fluorophores, unspecific labelling etc.), as well as physical models (bleaching, 2D and 3D PSF models) can be set and selected to provide realistic image data. A detailed comparison of the previous version of TestSTORM, SuReSim and the present version of TestSTORM can be found in the Supplementary material (Table S1).

The TestSTORM code was written in MatLab and can be easily handled via a GUI window (Figure S1). A detailed manual of the code can be downloaded from the homepage of the group^22^. In this paper the main features of TestSTORM are presented. The Supplementary Information together with the software manual contains all the additional relevant information.

### Gaussian versus scalar diffraction PSF models

**Figure 1:**
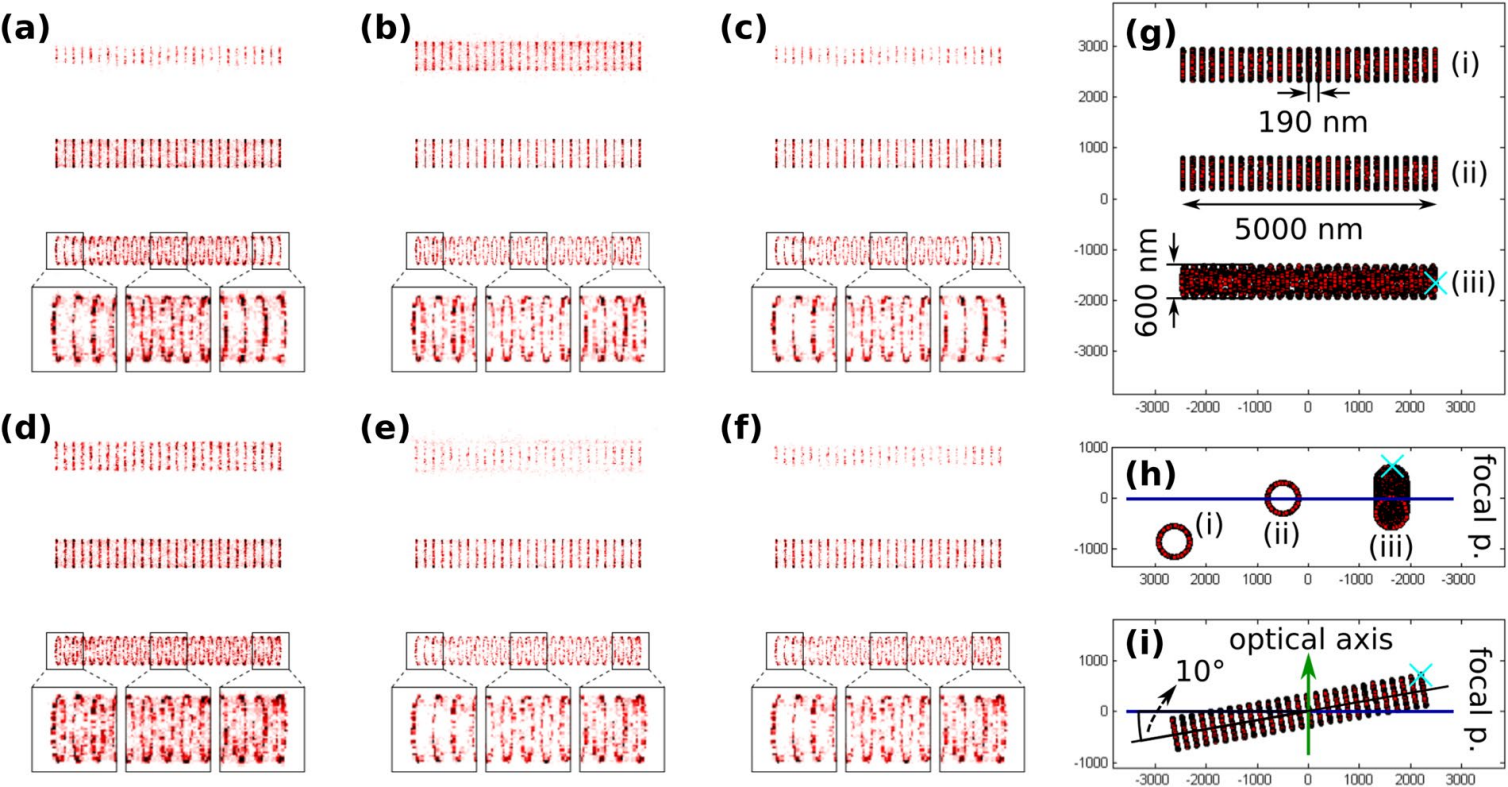
Simulated super-resolved dSTORM images with Gaussian-PSF (**a**–**c**), and with SD-PSF model (**c**–**e**) of the actin ring structure, and front (**g**) and side (**h**,**i**) views of the simulated actin ring patterns. Localizations were thresholded via their sigma (**a**,**d**) their residual (**b**,**e**) and both their sigma and residual values (**c**,**f**).

In localization based microscopy the 3D spatial distribution of the PSF is typically described by a Gaussian beam^23–26^. In this model the lateral distribution can be given by a Gaussian function (normal distribution) independently of the axial position (defocus) of the molecule. The intensity of the central peak decreases and the standard deviation (beam size) increases with the defocus but the overall Gaussian shape does not depend on the axial position. Localization algorithms can fit a Gaussian function to such a distribution and determine the position of the fluorescent molecule. The fitting parameters (peak intensity, standard deviation) depend on the axial position, and localization precision degrades with defocus, but localization is possible even under extreme defocus values. Application of such a Gaussian PSF model can lead to the misinterpretation of the final image. The real PSF shows a different profile^27^, the intensity drops down to zero on the optical axis and a ring system with high intensity side lobes characterizes the defocused PSF. Such features can be well described by optical diffraction theories^28, 29^. It is worth noting here, that due to the limited photon number in SMLM the PSF is typically undersampled. The standard deviation of the PSF was set to equal the pixel size of the CCD camera to maximize the localization precision, but this precluded the visualization and detection of the fine structures of the real PSF. Assuming a diffraction limited (optical aberration free) system the cylindrical symmetric Gaussian model works well, since the most critical parameter, the peak position does not depend on the PSF model. However, optical aberrations can distort the shape of the PSF and can slightly modify the peak positions and distort the final super-resolved image^30^. Therefore, a more realistic scalar diffraction PSF (SD-PSF) model was implemented based on the optical diffraction theory. A detailed description on the applied model can be found in the Supplementary Information (Figure S2). In order to examine the difference between the Gaussian and the SD-PSF models, simulations were performed using different axon segments^31, 32^ as shown in Fig. 1.

The axon pattern aims to mimic actin rings in axon initial segments. This pattern consists of equidistantly separated circles. The labels bond to epitopes that are placed on the outer surface of the rings with uniform distribution and with the given density. The density of epitopes is defined as the ratio of the number of epitopes on a circle and the circumference of the circle. By default the rings are separated by 190 nm and have radii of 300 nm; these are common values for axon initial segments^33, 34^. An axon pattern can consist of several segments whose axis orientations and center positions can be set. Figure 1g shows the arrangement of axon segments that were used for the comparison of the Gaussian and the SD-PSF models and for the simulation of the labelling density. The first segment is placed out of focus by −800 nm (Fig. 1h), the second (the middle one) is placed into the focal plane, and the third one is tilted at 10° (Fig. 1i). The image captured by the traditional fluorescent technique cannot reveal such a fine ring structure (Figure S4). Figure 1 depicts the reconstructed SMLM images under two different thresholding conditions. Localizations were ranked based on their residual (difference between the measured and the fitted Gaussian distribution) and their sigma (standard deviation of the fitted Gaussian) values. The ring system can be resolved by using either the Gaussian (Fig. 1a–c) or the SD-PSF (Fig. 1d–f) models and practically no difference can be visualized between them when the segment is placed in the focal plane (middle pattern). However, the reconstructed defocused and tilted structures (upper and bottom patterns) reveal different features. The image is more sensitive to the defocus using the Gaussian PSF model when localizations are only thresholded by their sigma values (Fig. 1a). Several 3D methods apply this feature^26, 35^ when the axial position of the fluorescent molecules is determined by the width of the measured intensity distribution. Due to the axial symmetry of the Gaussian beam, thresholding works identically in both defocus directions. On the other side, thresholding to the residual values does not introduce a serious sectioning effect, because a Gaussian beam has a Gaussian distribution in any defocused axial position. Therefore, the whole ring can be visualized on the tilted sample (insets in Fig. 1b). In contrast, the SD-PSF model is an asymmetric distribution, the shape of the PSF depends on the direction of the defocus (Figure S2). In this case thresholding to the residual values introduces an asymmetric image (Fig. 1e), and shows a stronger sectioning effect in the negative defocus direction when PSF has high intensity side lobes. In the other (positive) direction, thresholding to the sigma value shows only a moderate sectioning effect (Fig. 1d). It is interesting that when the above detailed two thresholding processes are applied simultaneously, the resulted images are very similar, independently of the used PSF models (Fig. 1c and f).

### Polarization dependent excitation and detection

**Figure 2:**
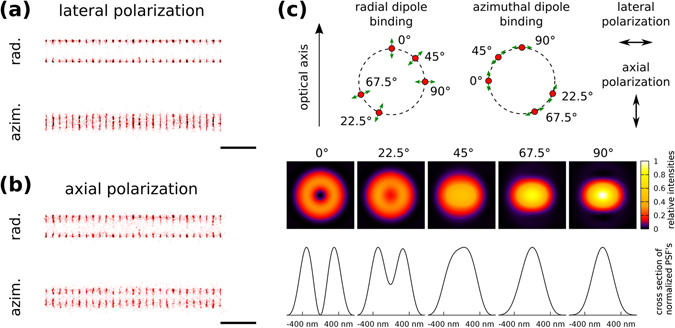
Polarization sensitive SMLM images of actin rings labelled by rigid fluorescent dyes with dipoles of radial and azimuthal orientations. Depending on the polarization of the excitation beam (**a**,**b**) dyes with specific orientations located at different regions on the rings can be excited. The acceptance of the localizations strongly depends on the shape of the PSF (**c**) determined by the relative position of the dye molecule to the optical axis. Scale bar is 1 micron.

In conventional SMLM the polarization effects are usually neglected. The samples are typically excited by a circularly polarized beam precluding photoselection or direction sensitive excitation. In many cases only the time averaged PSF can be detected because of the fast rotation of the fluorescent molecules^12^. However, even assuming rigid emission dipoles, the substructure of the polarization dependent PSF is still hindered due to undersampling. This is because maximum localization precision can be achieved when the standard deviation of the PSF approximately equals to the pixel size^10^. Despite the above mentioned issues, polarization sensitive SMLM is an active field of research, because polarization properties of single molecules strongly depend on the local environment (viscosity, FRET efficiency etc.) and hence can be used to monitor the close vicinity of the emitting dipole, or can provide additional information on the structure of the sample^36^. To simulate polarization dependent SMLM, an in-focus actin ring structure was imaged with rigidly labelled fluorescence dyes. Details on labelling model can be found in Supplementary Figure S5. Two special cases were studied, when the orientation of the dipoles were radial and azimuthal (Fig. 2c). In both cases dyes with absorption dipoles parallel to the polarization of the laser beam can be excited with higher propensity due to photoselection. Using lateral polarization excitation, parallel to the plane of the rings (Fig. 2a), dyes with an orientation of 90° (Fig. 2c) can be excited with the highest efficiency. Despite the elliptically distorted PSF (Fig. 2c) the peak position can be determined. Such a photoselection effect results in the split and narrowing of the structures with the radially and azimuthally oriented dyes, respectively. Using an excitation beam with longitudinal (axial) polarization, dyes with an orientation of 0° can be excited with the highest efficiency. Such an excitation scheme can be achieved via highly inclined illumination with a p-polarized beam^37^. In this case the captured PSF has a doughnut shape with an intensity minimum in its centre. Therefore, molecules that are excited with the highest propensity and emit the most photons cannot be fitted by a Gaussian distribution and hence cannot be localized. In contrast to the previous case, the SMLM images are split and narrowed in case of azimuthally and radially oriented dyes, respectively. General excitation and detection options at different dipole orientations are depicted in Supplementary Figure S3. In reality, when dipoles rotate during the exposure time, the final image is the weighted superposition of the above mentioned cases.

### Multicolour imaging

**Figure 3:**
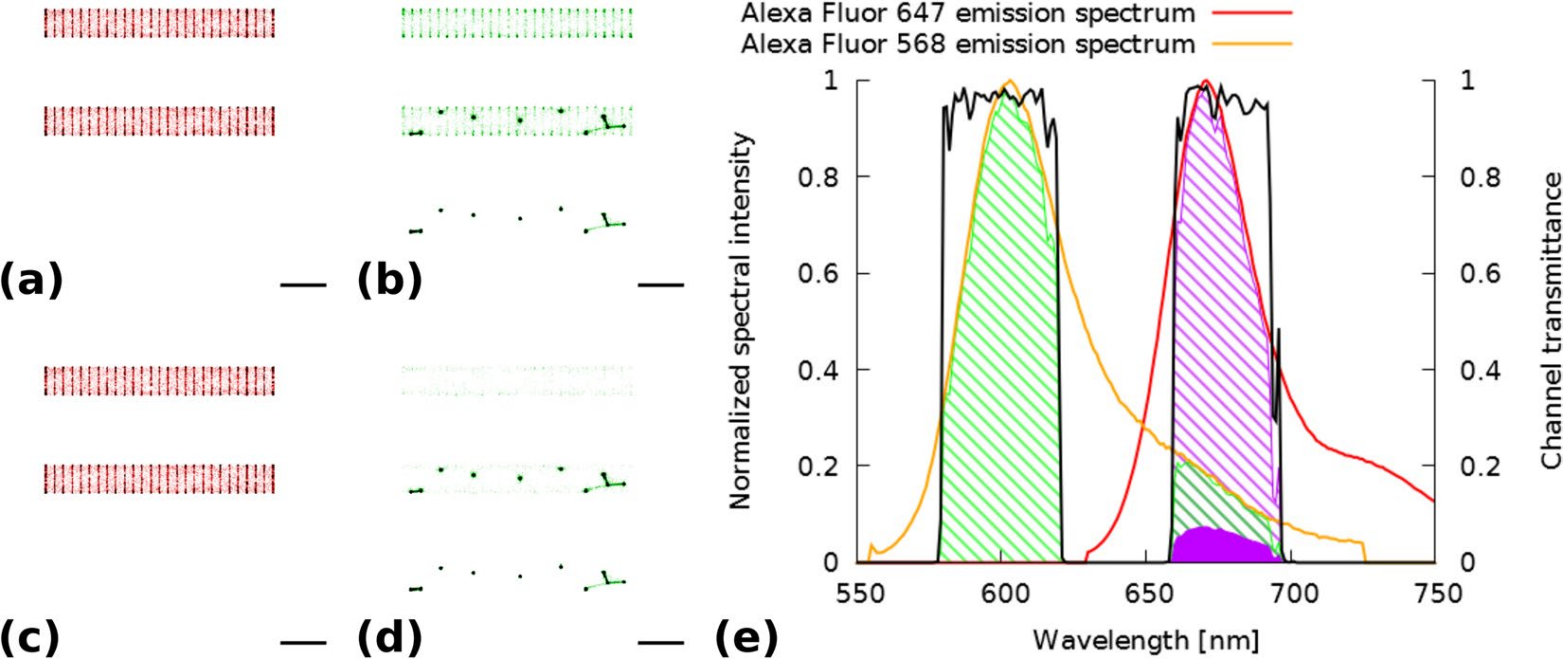
Spectral crosstalk between actin rings and vesicles labelled by Alexa 647 and Alexa 568 dyes, respectively, using a single multiband filter set and excitation of 647 nm (**a**,**c**) and 561 nm (**b**,**d**). Additional thresholding of the accepted localizations based on their photon number can reduce the crosstalk (**c**,**d**). Emission spectra of Alexa 568 (orange) and 647 (red) filtered by the multiband emission filter (black). Scale bar is 1 micron.

Spectral crosstalk originating either on the excitation (simultaneous excitation of different dyes due to the overlapping of their absorption spectra) or on the detection side (emission spectra overlap different transmission windows of the emission filter) is one of the most critical issues in fluorescent multicolour microscopy^38–42^. This issue is especially critical in SMLM when the insertion of additional spectral filters would reduce the detected photon number and hence degrade the localization precision. The microscope frames used for multicolour (d)STORM imaging are typically equipped with a multiband filter cube. In this paper a full-multiband filter set optimized for laser excitations of 405, 488, 561, & 635 nm (Semrock: LF405/488/561/635-A-000) was modelled. Figure 3 depicts the simulated super-resolved images of actin ring structures and vesicles, labelled by Alexa 647 and Alexa 568, respectively, under laser excitations of 647 nm and 561 nm. The actin rings can be seen clearly under excitation of 647 nm (Fig. 3a). In this case there is no crosstalk, since Alexa 568 dyes practically cannot be excited via the 647 nm laser. In contrast, at excitation of 561 nm, besides the labelled vesicles the actin rings can also be visualized (Fig. 3b), because Alexa 647 can be excited via the 561 nm laser and the emitted photons (see filled purple spectrum region in Fig. 3e) can reach the detector. However, the low excitation efficiency of Alexa 647 at 561 nm (7.5% of the maximum), results in a low photon number and hence in a low localization precision. By applying an additional higher threshold for photon number the vast majority of such imprecise localizations can be eliminated (Fig. 3d). Such tighter thresholding does not affect the image quality and hence it is unnecessary for the other channel (Fig. 3c). The spectral crosstalk between the two excitation channels can be eliminated via additional emission filters^43^. However, inserting and removing emission filters into the detection path in a reproducible way^44^ is a challenge. Such extra filters can be defined via their crosstalk values and used in TestSTORM under sequential or simultaneous laser excitations (Figure S6).

### Structured fluorescence background

**Figure 4:**
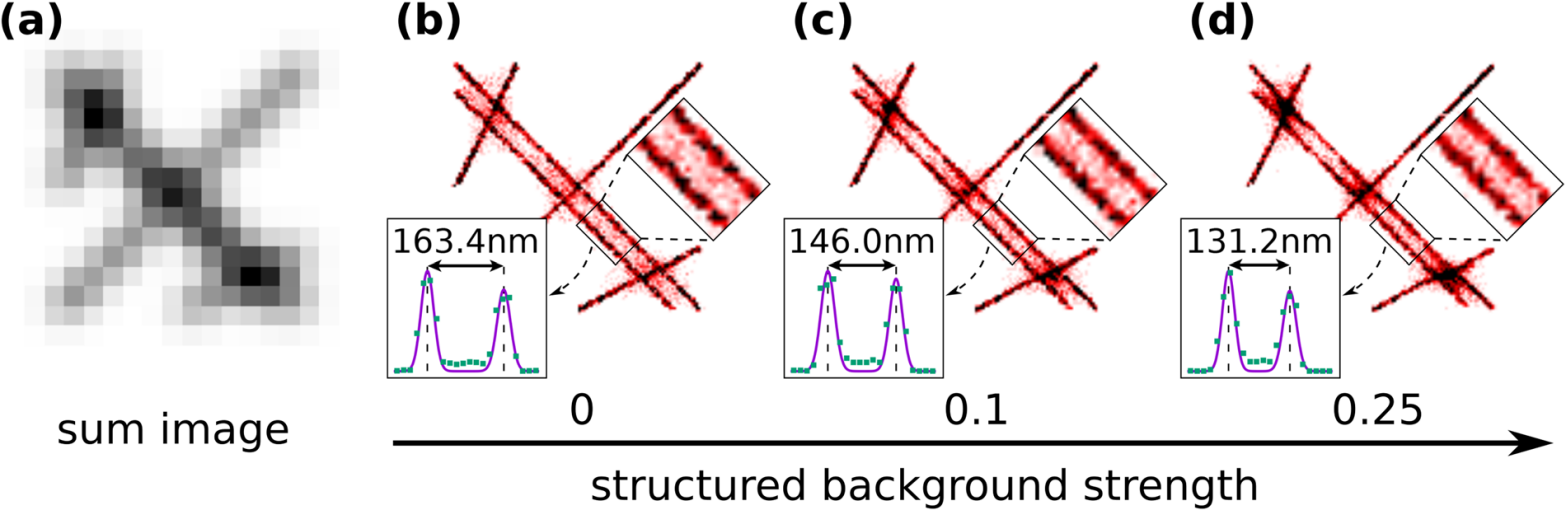
Conventional fluorescent (**a**) and SMLM images with different structured background levels (**b**–**d**). The higher the background level, the smaller the separation of the adjacent parallel lines. Insets show the separation of the main peaks of the fitted double Gaussian functions. Scale bar is 1 micron.

Localization precision strongly depends on the background noise level introduced either by the camera or the sample^10^. The fluorescent background generated by the sample can be also categorized into unstructured nonspecific labelling, structured autofluorescence and structured fluorescence background (SFB). Nonspecific labelling and autofluorescence can be reduced via the usage of appropriate labelling protocols, and spectral filtering strategies, respectively. However, SFB can be eliminated by neither of these techniques. SFB is especially critical in the widely used, highly inclined illumination mode when the excited depth in the sample is larger than the DOF (∝λ/NA^2^). Due to the Gaussian intensity distribution of the excitation beam the density of active fluorophores at the edge of the illuminated, but defocused region (i.e. below and above the imaged section) is high and results in structured fluorescence background. Such a background can also be simulated in TestSTORM and was examined using a line pattern (see Fig. 4) with different background levels. The structured fluorescent background is a weighted image of the sum image. The weighting value (structured fluorescent background strength) gives the intensity ratio of the brightest possible pixel of the localized dye molecule and the maximum pixel value of the structured background. The separation of the two adjacent parallel lines decreases with increasing SFB. The rate of line narrowing was found to be approximately 1 nm/%. Such a structure dependent artefact limits the quantitative evaluation of STORM images and their elimination requires advanced techniques and/or algorithms for more precise sectioning.

### Labelling density

The independent detection of fluorescent molecules is one of the most critical tasks in STORM imaging and requires a tight control of labelling density, i.e. the rate between on, off and bleached states^45^. In case of a high labelling density the diffraction spots (PSFs) can overlap, precluding single molecule detection. In contrast, at low labelling density undersampling can cause aliasing and leads to the misinterpretation of the final reconstructed image^30^. TestSTORM can reveal typical artefacts caused by inappropriate labelling density and find a trade-off between fast (overlapping PSFs) and slow (undersampled structure) image acquisition in an iterative way.

**Figure 5:**
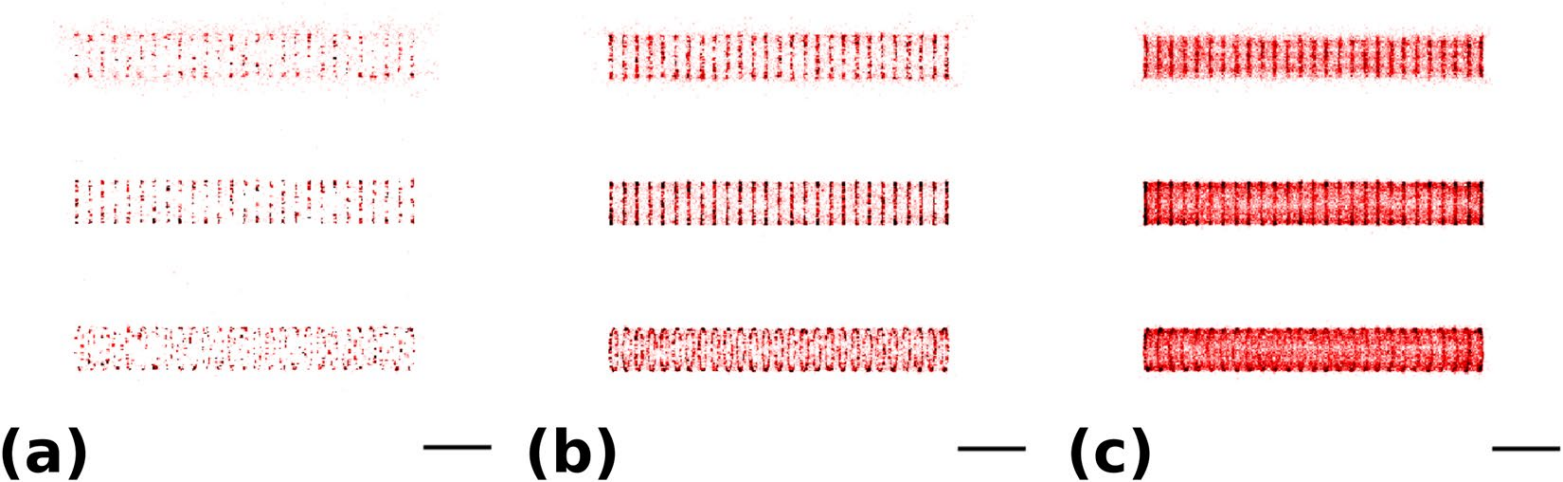
Axon segments at 10 dyes/μm (**a**), 50 dyes/μm (**b**) and 200 dyes/μm (**c**) labelling density values. Scale bar is 1 micron.

The effect of labelling density was examined using the very same axon segment structures as was depicted in Fig. 1. The reconstructed image at the lowest labelling density (10 labels/μm) did not result in sharp structures, the sample was undersampled (Fig. 5a). The 190 nm periodicity could be seen clearly only when the centre of the pattern was in focus, but even then the individual rings were inhomogeneous. When the labelling density was increased up to 50 dye/μm, the periodicity became clearly visible (Fig. 5b). By further increasing the labelling density, crosstalk between the rings starts playing an important role and degrades the image quality (Fig. 5c). It is worth noting that the width of the defocused pattern decreases with increasing labelling density. This is due to the fact that the 2D projection of a homogeneously labelled 3D cylinder is inhomogeneous and has a higher brightness at its rim. A brighter region means a higher chance of overlapping PSFs and results in less accepted localizations on the final high resolution image. The tilted sample depicts combined artefacts as a function of depth. This is probably the most realistic case, since axons are not perfect straight tubes oriented perpendicular to the optical axis of the microscope objective. During the simulations the precise alignment of the focus was an easy task. However, during real SMLM measurements focusing is a challenge and is only possible at a low excitation intensity when the whole structure is visible. In contrast, in the STORM imaging mode, when the excitation power is at around 1–50 kW/cm^2^, only the blinking molecules in the focus can be captured, but the structure cannot be visualized^5^.

### Drift simulation

Drift introduced by either thermal or mechanical reasons is a critical limitation of SMLM methods. Fiducial markers such as fluorescent beads or nanoparticles can be widely used to register frames to one other in the captured image stacks, however, they cannot be applied when a deeper tissue section is imaged.

**Figure 6:**
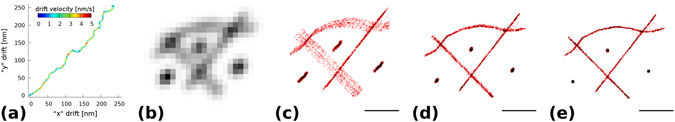
Drift trajectory and velocity (**a**) and its effects, (**b**): conventional image, (**c**): SMLM image without drift correction, (**d**) SMLM image with drift correction, (**e**) ground-true image without drift. Scale bar is 1 micron.

In TestSTORM drift is modelled with the movement of a point mass affected by random and drag forces in a medium flowing with the set mean drift velocity. Random forces alone would cause the drift velocity values to follow a random walk trajectory but the drag ensures that these values remain close to the given mean velocity. This kind of movement can be seen in Fig. 6a, the depicted drift changes both its velocity and direction over the frames but on large scale keeps its mean velocity.

A significant drift (1 nm/s in “x” and “y” directions) was applied to a pattern containing lines and vesicles. The lines were drawn with the JFilament ImageJ plugin^46^. The effects of such a drift and the corrected image (based on a blind drift correction algorithm implemented into rainSTORM) can be observed in Fig. 6c and d, respectively. These figures show that while the applied drift deforms the structure to a great extent, the correction algorithm negates the effects of the drift and the original structure can clearly be observed although it remains slightly more distorted than the localized image without any drift shown in Fig. 6e.

## Conclusions

A test sample simulator software was developed and tested for super-resolution localization microscopy. The implemented advanced methods made the simulated data more realistic, providing a more useful tool to optimize the critical imaging parameters and understand the origin of different imaging artefacts.

The axially symmetric Gaussian PSF was sensitive to the sigma value, but did not show any dependence on the residual value. In contrast, the asymmetric SD-PSF model showed direction dependent sensitivity to the sigma and residual values. Despite the significant difference between the two PSFs models, by applying appropriate thresholding processes the image quality of the final, high resolution images were found to be similar.

Taking into consideration the direction of the dipoles, polarization sensitive excitation and detection were also demonstrated using rings formed by actin filaments and labelled by rigid dye molecules. A significant difference between the captured images was realized under different dipole orientation and excitation conditions. Due to the rotation of the dipoles such characteristic features typically cannot be visualized, but using dyes rigidly bound to the target molecules anisotropy measurements at single molecule level are possible.

It was also shown that optimized spectral filtering can reduce spectral crosstalk between the channels and hence improve the multi-coloured image quality. Separation of the spectral channels makes the software capable of providing rough data for testing the different multicolour imaging techniques.

Image stacks with added structured background and/or mechanical drift of the sample can be also tested and used for the optimization of either the sample preparation protocol or the applied algorithm.

The above listed main features can be combined (just as they appear during a real measurement) and their effects can be evaluated and visualized by means of localization software.

## Electronic supplementary material

Supplementary Information
