# Supplementary material for: TestSTORM: Versatile simulator software for multimodal super-resolution localization fluorescence microscopy
Source: Sci Rep. 2017 Apr 19;7:951. doi: 10.1038/s41598-017-01122-7 (PMC5430448; doi:10.1038/s41598-017-01122-7)
Supplement: Supplementary file 1 — Supplementary Information [file 41598_2017_1122_MOESM1_ESM.pdf]

Supplementary information for “TestSTORM: Versatile simulator software for multimodal super-resolution localization fluorescence microscopy”

Tibor Novák[1], Tamás Gajdos[1], József Sinkó[1], Gábor Szabó[1,2] and Miklós Erdélyi[1]*

[1]Department of Optics and Quantum Electronics, University of Szeged, 6720 Szeged, Dóm tér 9, Hungary;

[2]MTA-SZTE Research Group on Photoacoustic Spectroscopy, Szeged, Hungary

*To whom correspondence should be addressed.

Supplementary Table S1| Comparison of test sample generator programs

Features	SuReSim	TestStorm v1.0	TestStorm v2.0
multi-colour measurement simulation	-	-	+
polarization sensitive measurement simulation	-	-	+
astigmatic 3D measurement simulation	+	-	+
seed control for random number generation	+	-	+
Gaussian PSF	+	+	+
scalar diffracted PSF	-	-	+
vector diffracted PSF and orientation dependent excitation	-	-	+
PSF anisotropy	-	-	+
predefined structures	+	+	+
arbitrary structures	+	-	+
combination of several structures in single simulation	+	-	+
visualization of dye molecules	+	-	+
visualization of structures	+	-	-
epitope density	+	+	+
multiple dye molecules on epitopes	+	-	+
binding angle distribution	+	-	+
non-specific labels	+	-	+
structured background	-	-	+
drift simulation	-	-	+
temporal trajectories of dye states	+	+	+
noise addition	+	+	+
acquisition parameters	+	+	+
simplified simulation of localization	+	-	-
TIFF stack output	+	+	+
dye positions file	+	+	+
save and load simulation parameters	+	-	+

Table S1. *Comparative table of features implemented into SuReSim*[1] *and into the first and the improved version of TestSTORM. (+: implemented features; -: not implemented features).*

Supplementary Figure S1| TestSTORM GUI

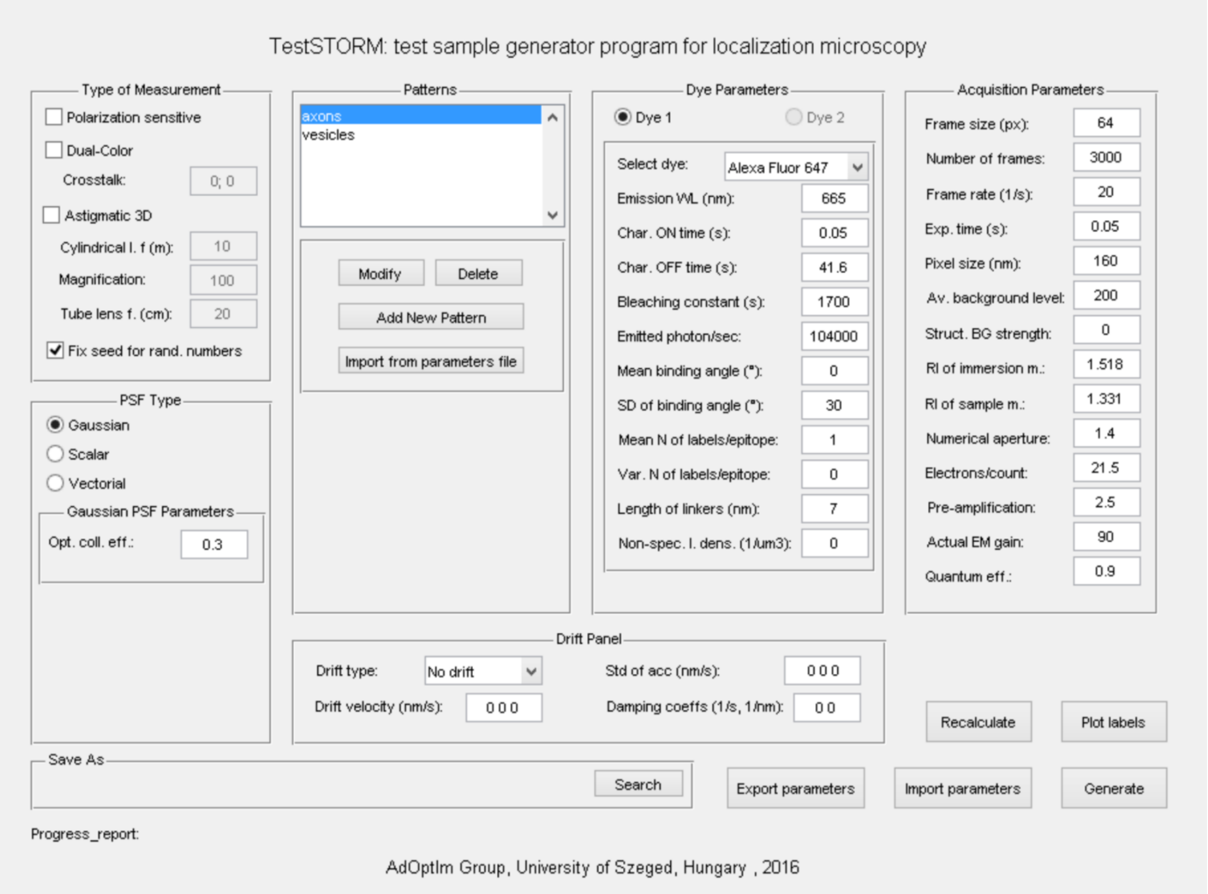

Figure S1. *Sample, dye and data acquisition parameters can be set by means of the GUI window of TestSTORM.*

In addition to "normal" SMLM simulations, TestSTORM can provide combined dual-colour, astigmatic 3D and polarization sensitive measurement simulations. The simulations can apply either the "random seed" or the "fix seed" random number generation processes. In the former process the sequence of blinking events varies from simulation to simulation, while in the latter one repeatable, identical image stacks are generated.

Multi-colour imaging is an important tool in fluorescence microscopy since it enables the observation of interactions and the relative spatial arrangement of sub-cellular structures[2-4]. In dual-colour simulations the structures can be labelled with two different dyes. During this simulation two image stacks are generated containing the PSFs of the first and the second dyes. Ideally, the first dye affects only the first image stack and the second dye affects only the second image stack, but in TestSTORM it is also possible to simulate the effects of crosstalk. The dye library contains the parameters of only two dyes, namely the Alexa Fluor

647 and the Alexa Fluor 568, although the user can define other dyes with arbitrary parameters.

Polarization sensitive measurements provide information about the fluorophore orientations, which are affected by the properties of the structure they are bonded to and by the local environment[5,6]. With polarization sensitive measurements it is possible to separate the PSFs created by the “x” and “y” components of the electric field incident on the image plane. With this measurement simulation mode only the vectorial PSF model can be used.

The astigmatic 3D measurement mode applies astigmatism to the PSFs which enables to obtain precise depth information about the sample[7]. In the Gaussian PSF model elliptical Gaussian intensity distributions are generated. The Rayleigh length is assumed to be the same in the “x” and “y” directions; it is only the beam waist positions that are shifted along the optical axis (“z” axis). This shift is determined by the focal point change in the “y” direction caused by the cylindrical lens. The zero position along the optical axis is defined as the position where the PSF widths of the two directions coincide between the two focal points. In the scalar or vector PSF models the phase front aberration of the cylindrical lens affects the PSF shape and the position of the circle of least confusion is predicted from the Gaussian model.

In addition to the Gaussian PSF model we also implemented scalar and vector diffraction based PSF models for more realistic PSF simulation. The scalar PSF model describes more realistically the dependence of the PSF size and shape on the defocus than the Gaussian model[8], while the vector model is used to simulate fixed dipole moments[9], and is also able to simulate PSF anisotropy[10]. In these PSF models the PSF size and shape can differ significantly from the Gaussian model, which affects the residual, the width (sigma value) and the central position[11] of the fitted Gaussian function. The computational cost of the scalar and vector PSF models is significantly higher than that of the Gaussian model. Generation of 100000 PSFs with the scalar model takes about 45-50 minutes with an old Intel(R) Pentium(R) D CPU 3.00GHz processor. The same calculation would take twice as much time using the vectorial PSF model without the PSF anisotropy, and would take more than six times longer with anisotropic PSFs.

Supplementary Figure S2| scalar diffraction PSF model

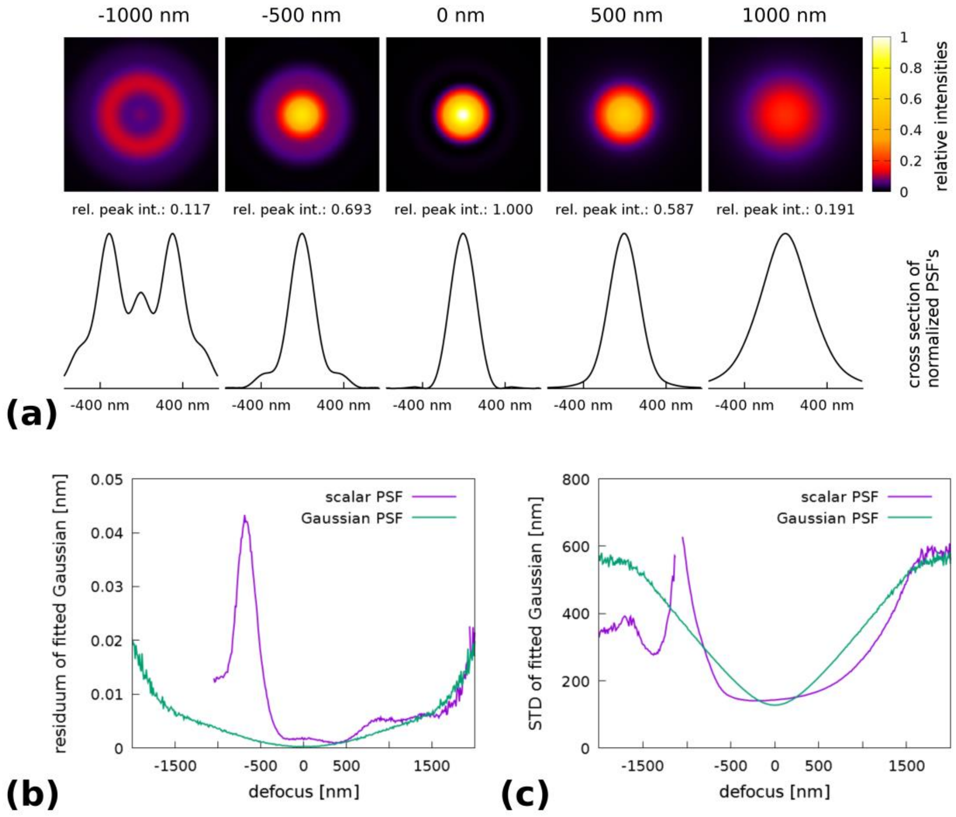

Figure S2. *Intensity distribution calculated via the scalar diffraction model using different defocus values, when the spherical wave front emitters were placed -1000 nm, -500 nm, 0 nm, 500 nm and 1000 nm out of focus (a). Comparison of the residual (b) and sigma (c) values of the fitting via the Gaussian and SD PSF models.*

The scalar diffracted PSFs are calculated using a modified Gibson and Lanni PSF model[12]. This PSF model includes the phase front aberration caused by the stratified medium, but we also included the transmission coefficients and the apodization function (these are also taken into account in the vectorial Richards and Wolf PSF model[13]). It is also assumed that the refractive index of the cover slip and that of the immersion medium are matched. However, the refractive index of the sample medium can be different. The immersion medium thickness, which affects the Gibson and Lanni phase aberration, is calculated by minimizing the variance value of the PSF originating from an emitter placed in the “zero” position. The effects of defocus and of the phase front tilt are calculated using Zernike polynomials[14].

The shape of the scalar diffracted PSF shows a strong and asymmetric dependence on the defocus value. We found that the applied localization algorithm is able to fit Gaussian

functions to the scalar diffracted PSFs with high confidence, except when extremely large defocus is used. Although the localized images of the Gaussian and scalar PSFs are similar, the residual and the width of the fitted Gaussians differ greatly as shown in Figure S2b-c. The scalar PSF model results in larger residuals for fitted Gaussians for most defocus values than the Gaussian PSF model. The scalar PSF model predicts larger PSF size in the focus than the Gaussian model, but its fitted sigma values are less sensitive to the defocus in the close vicinity of the focal plane. Consequently, thresholding and filtering with the sigma and residual values affect the localized image differently compared to the Gaussian model, as shown in Figure 1 in the paper.

Supplementary Figure S3| Photoselection and polarization sensitive detection

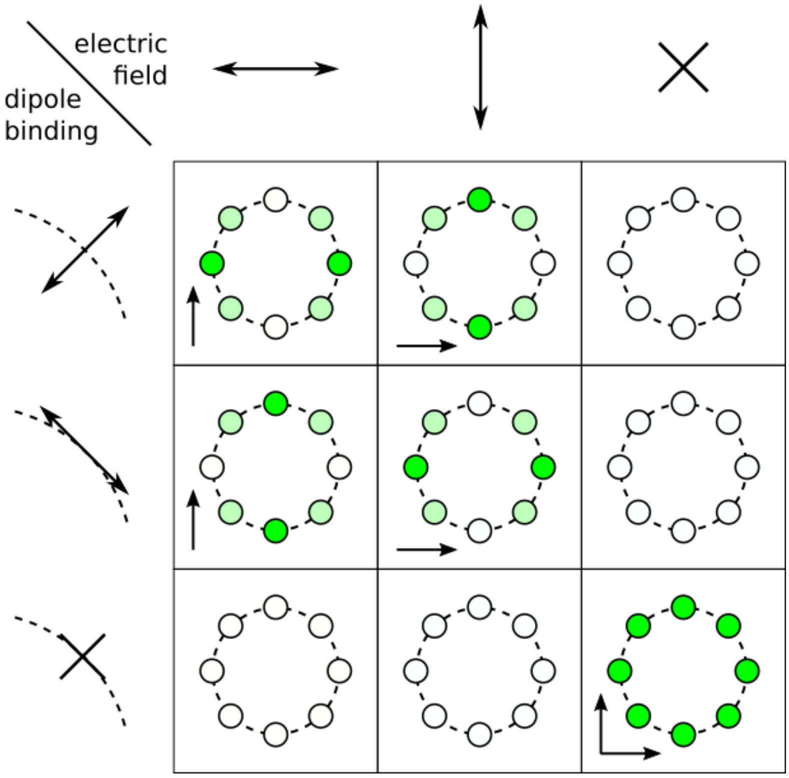

Figure S3. *Photoselection and polarization sensitive imaging of a ring labelled by the three orthogonal labelling and polarization directions of the fluorescent dye molecules and the excitation laser field vector, respectively.*

The vector diffracted PSFs are calculated using the Richards and Wolf PSF model[13]. This model takes into account the angular distribution of the dipole emitter's radiation as well as the aberrations caused by the stratified medium. In this model the shape and the intensity of the PSF shows strong dependence on the fluorophore orientation and the calculated intensity is highly dependent on the excitation field vector[15]. Consequently, the success and the accuracy of the localization of a blinking event depend on the excitation field direction, on the detection direction and also on the fluorophore orientation. When the dipole orientation is nearly perpendicular to the excitation field vector, the fluorophore is excited poorly and the low intensity may prevent the localization of the blinking event. When the dipole orientation is parallel to the optical axis, the PSF has a doughnut shape and the localization algorithm might not be able to fit the most commonly used Gaussian functions to it. These different cases are summarized in Figure S3. In the figure the green circles mark the position of dyes that can be excited with high propensity (polarization is parallel to the absorption dipole),

while the blank circles show the position of dyes with dipoles perpendicular to the excitation field vector, and hence cannot be excited. Arrows in the panels depict the ideal direction for localization, when the shape of the brightest blinking event's PSFs can be well estimated with a Gaussian function.

The vectorial PSF model implemented in TestSTORM is also able to take into account the effects of PSF anisotropy, which is useful for simulating flexibly bonded fluorophores. In PSF anisotropy simulations the dipole moment rotates freely and uniformly within a cone with a given cone angle[10]. The excitation strengths and the PSF shapes are averaged over the orientations within the cone. The averaging process also requires the ratio of the fluorescent lifetime and the rotational correlation time.

Supplementary Figure S4| Conventional fluorescent image of the actin ring system

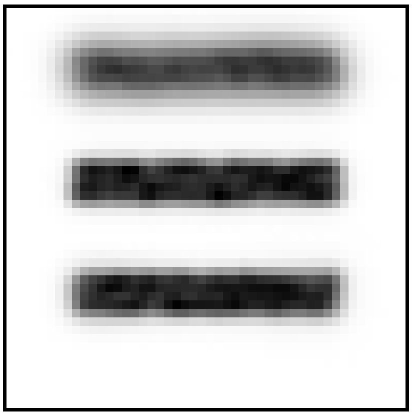

Figure S4. *Conventional fluorescent image of the actin ring pattern.*

Figure S4 depicts the sum image of the blinking events and it can be seen that conventional fluorescent imaging is unable to resolve the fine structure of the simulated axon segments. On the other hand the ring structures can clearly be observed in the localized image as shown in Figure 5b in the article.

Supplementary Figure S5| Labelling strategy

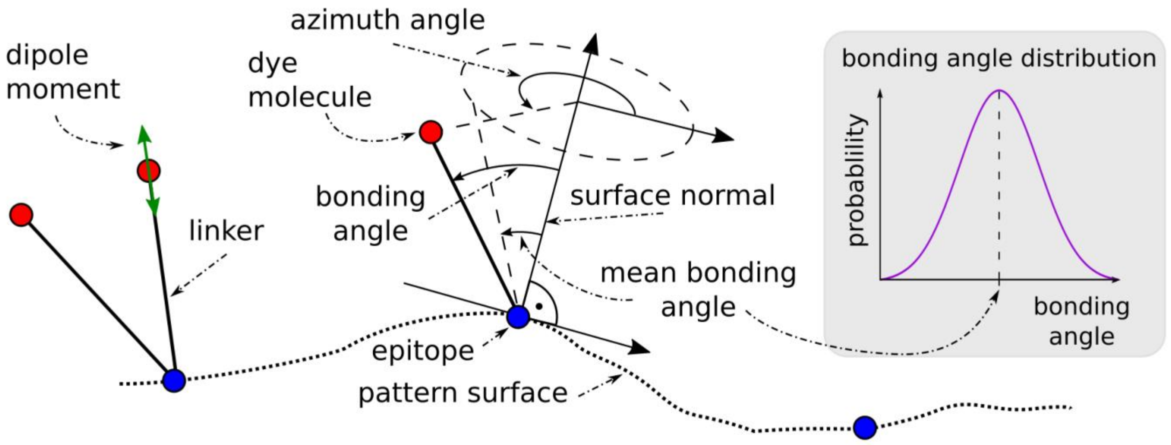

Figure S5. *Labelling strategy and geometry*

The labelling model in TestSTORM is intended to simulate the bonding of labels to epitopes in a simplified way. In the applied model the labels are placed on contact points serving as epitopes. The distance of the dye molecules and the contact points, the so-called "linker length", is fixed for all labels belonging to the same dye type. The number of labels placed on a single epitope follows binomial distribution characterized by itsmean and variance values. The angle between the linker and the surface normal is defined as the bonding angle. In case of point-like structures, i.e. array and octagon patterns, the fluorophores are placed uniformly on a sphere, while for other structures the epitope density on the spheresfollows normal distribution characterized by the given mean angle and standard deviation. In case of axon or line patterns the vector pointing to the maximum of the bonding angle distribution is in the plane defined by the surface normal and by the tangent vector of the ring or of the line. In case of star, vesicle or meshpatterns the bonding angle distribution does not possess a single peak, but has rotational symmetry around the surface normal. In case of vectorial PSF calculation the dipole orientation is assumed to be parallel with the linker vector.

Supplementary Figure S6 | Spectral filtering

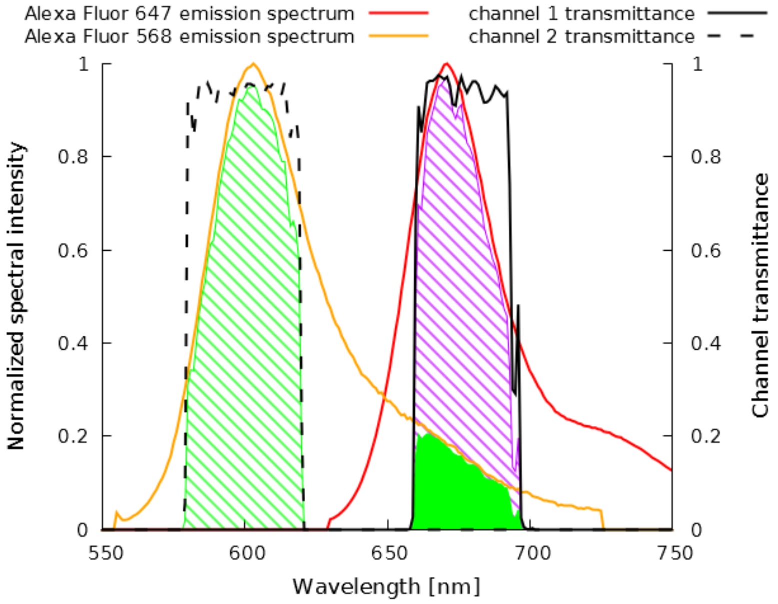

Figure S6. *Normalized emission spectra of Alexa Fluor 568 and Alexa Fluor 647 dyes filtered by the two bandpass filters.*

In dual-colour measurement simulations one can place different types of dyes on two adjacent structures. In addition to the described simulation in the paper it is possible to perform the simulation by simultaneously exciting the two dyes and separating the emission spectra with a dichroic mirror[16]. In such a case crosstalk is directly shown in Figure S6. We defined the crosstalk of the Alexa Fluor 568 as the ratio of power transmitted through the first channel and the power transmitted through both channels. This is shown in the figure as the ratio of the green filled area and the sum of the green filled and the green lined areas, which gives a 14.7% crosstalk value in this case. The Alexa Fluor 647 has nearly zero crosstalk because the emission spectrum of this dye and the transmission spectrum of the second channel overlap negligibly.

Supplementary Figure S7| Spectral filtering

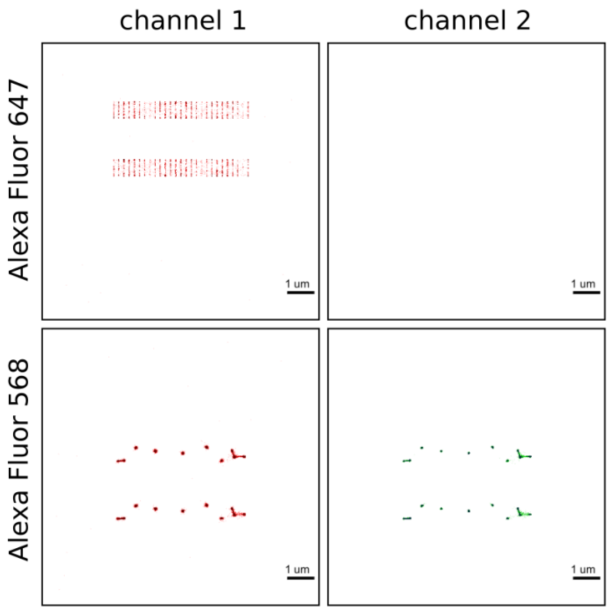

Figure S7. *SMLM images of the ring system and vesicles labelled by Alexa 647 and Alexa 568, respectively. The photons emitted by the sequentially excited dyes (with lasers operating at 647 nm and 561 nm) were spectrally separated into two spectral channels (channel 1: 647 nm excitation; channel 2: 561 nm excitation). Significant crosstalk can only be obtained via detection in channel 1 (spectral channel for Alexa 647) under the excitation of the Alexa 568 dye.*

Figure S7 shows the localized images when the Alexa 568 and the Alexa 647 dyes are excited separately, and the emission spectra of both dyes are captured in both colour channels. This situation clearly shows how the two different dyes affect the images captured in the two channels. The Alexa 647 has negligible crosstalk, consequently it appears only in the first colour channel. In contrast, the Alexa Fluor 568 has a large, 14.7% crosstalk as shown in Figure S6, and its blinking events can be localized in images captured by both channels. Since the Alexa Fluor 568's photon number is much lower in the first colour channel than in the second one, the vesicles are more distorted in the first channel due to the lower localization accuracy. This crosstalk can be eliminated via thresholding the photon number as shown in Figure S8, and the two structures can clearly be separated even in the case of simultaneous excitation.

Supplementary Figure S8| Spectral filtering

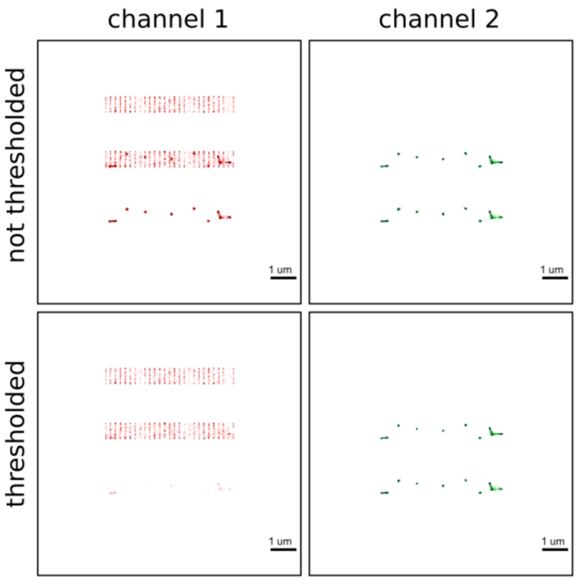

Figure S8. *SMLM images of the ring system and vesicles labelled by Alexa 647 and Alexa 568, respectively. The photons emitted by the simultaneously excited dyes (with lasers operating at 647 nm and 561 nm) were spectrally separated into two channels (channel 1: 647 nm excitation; channel 2: 561 nm excitation). Additional thresholding to the localization precision (using the photon number) reduced the spectral crosstalk and hence improved the final image quality.*

References

1. Venkataramani, V., Herrmannsdörfer, F., Heilemann, M., Kuner, T. SuReSim: simulating localization microscopy experiments from ground truth models. *Nat. Methods* **13,** 319-21 (2016).
2. Tam, J., Cordier, G. A., Borbely, J. S., Álvarez, Á. S., Lakadamyali, M. Cross-Talk-Free Multi-Color STORM Imaging Using a Single Fluorophore. *PLoS One* **9,** 111878; 10.1371/journal.pone.0111878 (2014).
3. Bates, M., Dempsey, G. T., Chen, K. H., Zhuang, X. Multicolor Super-Resolution Fluorescence Imaging via Multi-Parameter Fluorophore Detection. *ChemPhysChem.* **13,** 99-107 (2012).
4. Bates, W. M., Huang, B., Dempsey, G. T., Zhuang, X. Multicolor Super-resolution Imaging with Photo-switchable Fluorescent Probes. *Science* **317,** 1749–1753 (2007).
5. Erdelyi, M., Simon, J., Barnard, E. A., Kaminski, C. F. Analyzing receptor assemblies in the cell membrane using fluorescence anisotropy imaging with TIRF microscopy. *PLoS One* **9,** 100526 10.1371/journal.pone.0100526 (2014).
6. Backer, A. S., Lee, M. Y., Moerner, W. E. Enhanced DNA imaging using super-resolution microscopy and simultaneous single-molecule orientation measurements. *Optica* **3,** 3-6 (2016).
7. Kao, H. P., Verkman, A. S. Tracking of single fluorescent particles in three dimensions: use of cylindrical optics to encode particle position. *Biophys. J.* **67,** 1291-300 (1994).
8. Kirshner, H., Aguet, F., Sage, D., Unser, M. 3-D PSF fitting for fluorescence microscopy: implementation and localization application. *J. Microsc.* **249,** 13–25 (2013).
9. Aguet, F., Geissbühler, S., Märki, I., Lasser, T., Unser, M. Super-resolution orientation estimation and localization of fluorescent dipoles using 3-D steerable filters. *Opt. Express* **17,** 6829-48 (2009).
10. Backer, A. S., Lee, M. Y., Moerner, W. E. Enhanced DNA imaging using super-resolution microscopy and simultaneous single-molecule orientation measurements: supplementary material. *Optica* **3,** 000659; 10.1364/OPTICA.3.000659.s001 (2016).
11. Enderlein, J., Toprak, E., Selvin, P. R. Polarization effect on position accuracy of fluorophore localization. *Opt. Express* **14,** 8111-20. (2006).
12. Gibson, S. F., Lanni, E. Experimental test of an analytical model of aberration in an oil-immersion objective lens used in three-dimensional light microscopy. *J. Opt. Soc. Am. A.* **9,** 154-66 (1992).

13. Richards, B., Wolf, E., "Electromagnetic diffraction in optical systems—II. Structure of the image field in an aplanatic system. *Proc. R. Soc*. **253,** 358–379 (1959).
14. Mahajan, V. N. Zernike circle polynomials and optical aberrations of systems with circular pupils. *Appl. Opt.* **33,** 8121 (1994).
15. Stallinga, S., Rieger, B. Accuracy of the Gaussian Point Spread Function model in 2D localization microscopy. *Opt. Express* **18,** 24461-76 (2010).

16. Baddeley, D. et al. 4D super-resolution microscopy with conventional fluorophores and single wavelength excitation in optically thick cells and tissues. *PLoS One* **6,** 20645; 10.1371/journal.pone.0020645. (2011).
